# Fortilin inhibits p53, halts cardiomyocyte apoptosis, and protects the heart against heart failure

**DOI:** 10.1038/s41420-021-00692-w

**Published:** 2021-10-23

**Authors:** Preedakorn Chunhacha, Decha Pinkaew, Patuma Sinthujaroen, Dawn E. Bowles, Ken Fujise

**Affiliations:** 1 Division of Cardiology, Department of Medicine, University of Washington, Seattle, WA 98109 USA; 2 Division of Surgical Sciences, Department of Surgery, Duke University, Durham, NC 27710 USA; 3 Present Address: Department of Biochemistry and Microbiology, and Cell-based Drug and Health Product Development Research Unit (CDD), Faculty of Pharmaceutical Sciences, Chulalongkorn University, Bangkok, 10330 Thailand; 4 Present Address: Department of Pathology, Faculty of Medicine, Prince of Songkla University, Songkhla, 90110 Thailand

**Keywords:** Cardiomyopathies, Cardiomyopathies

## Abstract

Heart failure (HF) has reached epidemic proportions in developed countries, affecting over 20 million people worldwide. Despite modern medical and device therapies, 60–70% of HF patients still die within 5 years of diagnosis as it relentlessly progresses through pervasive apoptotic loss of cardiomyocytes. Although fortilin, a 172-amino-acid anti-p53 molecule, is one of the most expressed proteins in the heart, its precise role there has remained unknown. Also unclear is how cardiomyocytes are protected against apoptosis. Here, we report that failing human hearts express less fortilin than do non-failing hearts. We also found that mice lacking fortilin in the heart (fortilin^KO-heart^) die by 9 weeks of age due to extensive cardiomyocyte apoptosis and severe HF, which suggests that fortilin sustains cardiomyocyte viability. The lack of fortilin is also associated with drastic upregulation of p53 target genes in the hearts. The heart-specific deletion of p53 in fortilin^KO-heart^ mice extends their life spans from 9 to 18 weeks by mitigating cardiomyocyte apoptosis. Our data suggest that fortilin is a novel cardiac p53 inhibitor and that its inadequate expression in failing hearts and subsequent overactivation of the p53 apoptosis pathway in cardiomyocytes exacerbates HF.

## Introduction

Heart failure (HF)—a complex clinical syndrome secondary to structural and functional impairments of the heart muscle—has reached epidemic proportions in developed countries, currently affecting over 20 million people worldwide. Despite modern medical and device therapies, 30–40% of HF patients die within 1 year and 60–70% die within 5 years of diagnosis [1]. Although coronary artery disease and hypertension are the two most common causes of HF [2], HF progresses even after they are adequately treated, as biological changes initiated in failing human cardiomyocytes continue to expand [3]. These changes include myocyte hypertrophy, desensitization of β-adrenergic signaling, changes in excitation–contraction coupling, progressive loss of myofilaments within cardiomyocytes, and, most importantly, gradual and irreversible apoptotic loss of cardiomyocytes [3–7]. Although prevention of cardiomyocyte apoptosis could slow the progression of HF, how apoptosis is regulated in the heart is unclear.

Fortilin (also known as translationally controlled tumor protein, histamine-releasing factor, and TPT1) is a highly conserved, 172-amino-acid, 20 kDa protein that blocks apoptosis [8–10], and it is one of the most expressed genes in the heart [11]. Fortilin directly binds to and negatively regulates the tumor suppressor protein p53 [12, 13] and the endoplasmic reticulum (ER)-stress handling protein IRE1α [14], both of which promote loss of cardiomyocytes and subsequent HF [15–17]. Despite its abundance, the exact role of fortilin in the heart is poorly understood. Here we present evidence that fortilin is a major negative regulator of p53 in the heart and that its lack leads to massive apoptotic loss of cardiomyocytes and lethal HF.

## Results

### Human HF and fortilin

**Fig. 1:**
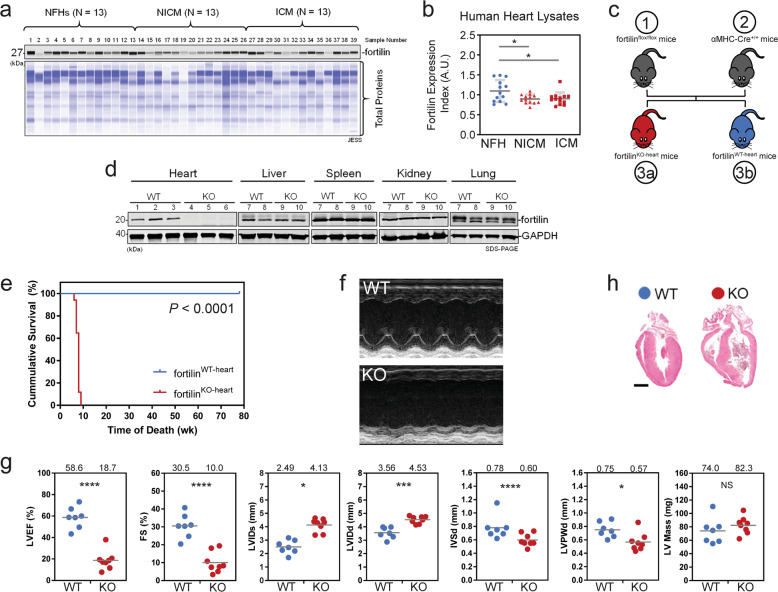
Mice lacking fortilin in the heart died prematurely due to severe systolic heart failure. **a**, **b** Fortilin expression levels were lower in the hearts of patients with NICM and ICM than in those of NFH patients. **c** A strain of mice lacking fortilin in the cardiomyocytes (fortilin^KO-heart^) (3a) and its control (fortilin^WT-heart^) (3b) were generated by crossing fortilin^flox/flox^ (1) and αMHC-Cre^+/+^ mice (2). **d** Fortilin protein levels were drastically lower in the hearts of fortilin^KO-heart^ mice than in those of fortilin^WT-heart^ mice. **e** All fortilin^KO-heart^ mice died by 9 weeks of age. **f**, **g** Transthoracic echocardiography at 7 weeks of age revealed dilated, thinned, and severely dysfunctional left ventricle in fortilin^KO-heart^ mice. **h** Sagittal heart sections of fortilin^WT-heart^ and fortilin^KO-heart^ mice. NFH non-failing hearts, NICM non-ischemic cardiomyopathy, ICM ischemic cardiomyopathy, JESS an automated capillary-based quantitative Western blot system with in-capillary total protein evaluation capability, A.U. arbitrary units, WT fortilin^WT-heart^ (or αMHC-Cre^−/−^fortilin^flox/flox^) mice that express fortilin normally in the heart, KO fortilin^KO-heart^ (or αMHC-Cre^+/+^fortilin^flox/flox^) mice that do not express fortilin in the heart, GAPDH glyceraldehyde 3-phosphate dehydrogenase, LVEF left ventricular (LV) ejection fraction, FS fractional shortening, LVIDs LV internal diameters in systole, LVIDd LV internal diameters in diastole, IVSd interventricular septum thickness in diastole, LVPWd LV posterior wall thickness in diastole. Scale bar = 500 µm; Error bars, means ± SD, statistical analyses performed using Student’s two-sample *t*-test, NS not statistically significant; *P < 0.05; ***P < 0.005; ****P < 0.001 (see also Fig. S1).

To evaluate the role of fortilin in the heart and in human HF, we subjected tissue lysates of human hearts from subjects with non-failing hearts (NFHs) and HF patients with non-ischemic cardiomyopathy (NICM) and ischemic cardiomyopathy (ICM) from the Duke Human Heart Repository [18] to an automated capillary-based quantitative Western blot analyses (JESS™, Protein Simple) [19, 20] (Fig. 1a). We calculated a fortilin expression index by dividing the area under the curve of a fortilin peak by the total proteins loaded in the same capillary. We found that fortilin expression was significantly lower in NICM and ICM hearts than in NFHs (Fig. 1b).

### Heart-specific fortilin-knockout (KO) mice

To test the hypothesis that fortilin deficiency in the heart leads to HF, we first generated fortilin^flox/flox^ mice using standard homologous recombination techniques [21]. We then generated heart-specific *fortilin* KO mice (αMHC-Cre^+/+^fortilin^flox/flox^, referred to here as fortilin^KO-heart^ mice) by crossing fortilin^flox/flox^ mice with mice over-expressing Cre-recombinase under the control of the cardiac-specific *Myh6* promoter [22] (αMHC-Cre^+/+^, Jackson Laboratory) (Fig. 1c). In cardiomyocytes of fortilin^KO-heart^ mice, the fortilin genomic sequence flanked by the LoxP sequences (Fig. S1a-1) is excised by the tissue-specifically expressed Cre-transgene (Figs. S1a-2, S1a-3a), while fortilin is normally expressed in all other tissue (Fig. S1a-3b). We used αMHC-Cre^−/−^fortilin^flox/flox^ (fortilin^WT-heart^) mice as the control. As expected, fortilin was not detectable in the hearts of fortilin^KO-heart^ mice, whereas it was normally expressed in all other tissues at both message (Fig. S1b) and protein (Fig. 1d) levels.

Although apparently normal at birth and fertile, fortilin^KO-heart^ mice started to die as early as 6 weeks of age and all were dead by 9 weeks of age (Fig. 1e). We performed transthoracic echocardiography on the surviving mice at 7 weeks of age (Fig. 1f) and found that the hearts were (i) dilated (increased left ventricular (LV) internal diameter in systole (LVIDs) and increased LV internal diameter in diastole (LVIDd)), (ii) thinned (decreased interventricular septum thickness in diastole (IVSd) and decreased LV posterior wall thickness in diastole (LVPWd)), and (iii) severely dysfunctional (decreased LV ejection fraction (LVEF) and decreased fractional shortening (FS) [%]) (Fig. 1g), consistent with the gross examination of the hearts of fortilin^WT-heart^ and fortilin^KO-heart^ mice (Fig. 1h). In addition, the ratio of heart weights to body weights (HW/BW) and that of lung weights to body weights (LW/BW) of fortilin^KO-heart^ mice were significantly greater than those of fortilin^WT-heart^ mice at 8 weeks of age (Fig. S1c), suggesting the presence of cardiomegaly and lung congestion, respectively, in fortilin^KO-heart^ mice. Further, RT-qPCR assays on RNAs from the hearts revealed the upregulation of heart-failure genes [23, 24] (*Col1*, *Myh7*, and *Anf*) in fortilin^KO-heart^ mice, but not in fortilin^WT-heart^ mice (Fig. S1d).

**Fig. 2:**
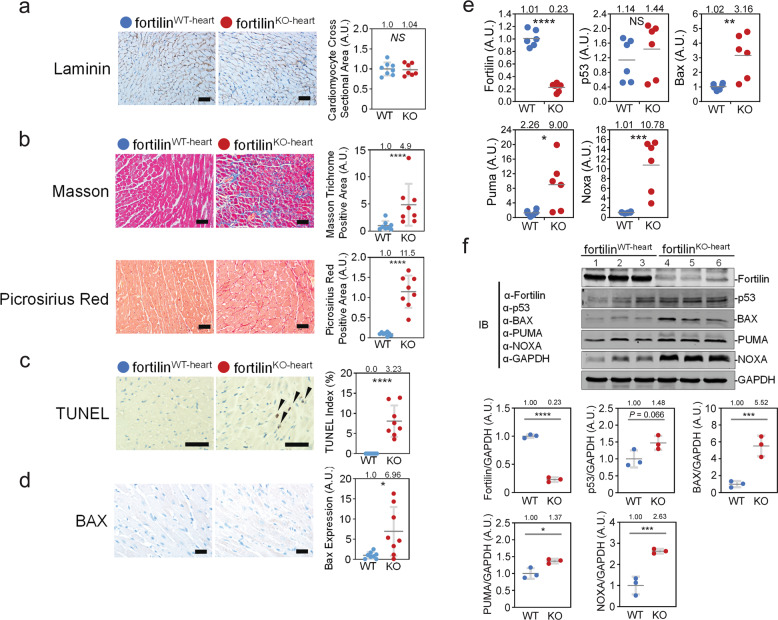
Severe fibrosis and apoptosis were evident in the hearts of fortilin^KO-heart^ mice, with signs of activation of the p53 pathway. **a** The size of cardiomyocytes was similar in fortilin^WT-heart^ and fortilin^KO-heart^ mice, as shown in laminin staining. **b** Both Masson and picrosirius red staining showed severe fibrosis in the heart of fortilin^KO-heart^ mice. **c** TUNEL staining revealed a significantly higher frequency of apoptotic cardiomyocytes in fortilin^KO-heart^ hearts compared to fortilin^WT-heart^ hearts. **d** BAX immunogenicity was drastically greater in fortilin^KO-heart^ than in fortilin^WT-heart^ mice. **e** The message levels of the p53-target, pro-apoptotic genes *BAX*, *PUMA*, and *NOXA* were significantly greater in the hearts of fortilin^KO-heart^ mice than in those of fortilin^WT-heart^ mice according to RT-qPCR assays of the heart RNAs. **f** The protein levels of BAX, PUMA, and NOXA were significantly greater in the hearts of fortilin^KO-heart^ mice than in those of fortilin^WT-heart^ mice, as shown by Western blot analyses of the heart lysates. A.U. arbitrary units, WT fortilin^WT-heart^ (or αMHC-Cre^−/−^fortilin^flox/flox^) mice that express fortilin normally in the heart, KO fortilin^KO-heart^ (or αMHC-Cre^+/+^fortilin^flox/flox^) mice, TUNEL terminal deoxynucleotidyl transferase dUTP nick-end labeling to identify apoptotic cells; scale bar = 100 µm; IB immunoblot, α-fortilin anti-fortilin antibody, GAPDH glyceraldehyde 3-phosphate dehydrogenase. Error bars means ± SD, statistical analyses performed using Student’s two-sample *t*-test: NS not statistically significant; *P *<* 0.05; **P *<* 0.01; ***P < 0.005, ****P < 0.001.

The size of cardiomyocytes did not differ between fortilin^WT-heart^ and fortilin^KO-heart^ mice (Fig. 2a), but both Masson and picrosirius red staining showed severe fibrosis in the hearts of fortilin^KO-heart^ mice (Fig. 2b). Terminal deoxynucleotidyl transferase dUTP nick-end labeling (TUNEL) revealed a drastically higher frequency of apoptotic cardiomyocytes in fortilin^KO-heart^ hearts compared to fortilin^WT-heart^ hearts (Fig. 2c). Additionally, the immunogenicity of the apoptosis inducer BAX was seven times greater in fortilin^KO-hearts^ than in fortilin^WT-hearts^ (Fig. 2d).

Chen et al. reported that fortilin binds to the sequence-specific DNA binding domain of p53, which blocks p53-induced transcriptional activation of BAX [12]. To test the status of activation of *BAX*, *PUMA*, and *NOXA*, which are all p53-target, pro-apoptotic genes, in the hearts of fortilin^KO-heart^ mice, we subjected the hearts to both RT-qPCR and Western blot analyses. All three genes were significantly more expressed in the hearts of fortilin^KO-heart^ mice than in those of fortilin^WT-heart^ mice at both message (Fig. 2e) and protein (Fig. 2f) levels, suggesting that the lack of fortilin in cardiomyocytes may lead to p53-induced transcriptional activation of these genes, leading to cardiomyocyte apoptosis and myocardial fibrosis. The results of these processes are severe HF and death of fortilin^KO-heart^ mice.

### Lack of p53 improved survival

**Fig. 3:**
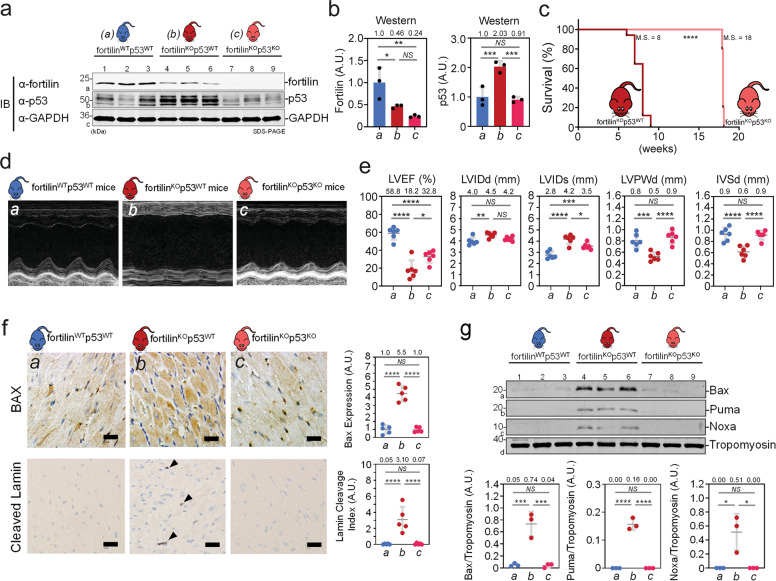
Lack of p53 partially rescued the premature death of fortilin^KO-heart^ mice. **a**, **b** Western blot analyses show that fortilin protein levels were significantly lower in fortilin^KO^p53^WT^ and fortilin^KO^p53^KO^ mice than in fortilin^WT^p53^WT^ mice. p53 protein levels were significantly lower in fortilin^KO^p53^KO^ mice than in fortilin^KO^p53^WT^ mice. **c** The lack of p53 in the heart of fortilin^KO-heart^ mice increased the M.S. from 8 to 18 weeks. **d**, **e** Echocardiography revealed that the hearts of fortilin^KO^p53^KO^ mice had better overall heart function than those of fortilin^KO^p53^WT^ mice, as evidenced by greater LVEF, less LVIDs (less cavity dilatation), and greater IVSd and LVPWd (less wall thinning). **f** Immunohistochemistry showed that the lack of p53 drastically reduced the expression levels of both BAX and cleaved lamin, an apoptosis marker. **g** Western blot analyses showed that the expression levels of BAX, PUMA, and NOXA were significantly decreased when p53 was deleted from fortilin-deficient hearts. *a* fortilin^WT^p53^WT^ mice (αMHC-Cre^−/−^fortilin^flox/flox^p53^flox/flox^ mice), *b* fortilin^KO^p53^WT^ mice (αMHC-Cre^+/+^fortilin^flox/flox^p53^WT/WT^ mice), *c* fortilin^KO^p53^KO^ mice (αMHC-Cre^+/+^fortilin^flox/flox^p53^flox/flox^ mice), IB immunoblot, α-fortilin anti-fortilin antibody, GAPDH glyceraldehyde 3-phosphate dehydrogenase, A.U. arbitrary unit, M.S. median survival, LVEF left ventricular (LV) ejection fraction, LVIDs LV internal diameters in systole, LVIDd LV internal diameters in diastole, LVPWd LV posterior wall thickness in diastole, IVSd interventricular septum thickness in diastole. Scale bar = 200 µm; Error bars, means ± SD, statistical analyses performed using ANOVA with Fisher’s multiple comparison except for the survival assay, for which the Log-Rank (Mantel–Cox) test was used: NS not statistically significant; *P < 0.05; **P < 0.01; ***P *<* 0.005, ****P < 0.001 (see also Figs. S2 and S3).

To test the hypothesis that the lack of fortilin causes the heart to fail through overactivation of the p53 apoptotic pathway, we knocked out ﻿p53 by crossing fortilin^KO-heart^ mice with p53^flox/flox^ mice [25] and generated fortilin^KO-heart^p53^KO-heart^ and fortilin^WT-heart^p53^KO-heart^ mice (Fig. S2a and S2b). Fortilin expression levels were significantly higher in fortilin^WT-heart^p53^WT-heart^ mice (fortilin^WT^p53^WT^, hereafter) than in fortilin^KO^p53^WT^ and fortilin^KO^p53^KO^ mice (the latter two had comparable expression), both at the message (Fig. S2c, fortilin, *a* vs. *b* and *a* vs. *c*) and protein (Fig. 3a and b, fortilin, *a* vs. *b* and *a* vs. *c*) levels. In addition, total p53 expression levels were significantly higher in fortilin^KO^p53^WT^ than in fortilin^KO^p53^KO^ mice, both at the message (Fig. S2c, p53, *b* vs. *c*) and protein levels (Fig. 3a and b, p53, *b* vs. *c*; Fig. S3a, p53, *b* vs. *c*). p53 expression levels also were significantly higher in fortilin^KO^p53^WT^ than in fortilin^WT^p53^WT^ mice, both at the message (Fig. S2c, p53, *a* vs. *b*) and protein levels (Fig. 3b, p53, *a* vs. *b*; Fig. S3a, p53, *a* vs. *b*). On the other hand, phosphorylated p53, an active form of p53, was most abundant in the hearts of fortilin^KO^p53^WT^ mice, followed by those of fortilin^WT^p53^WT^ and then by those of fortilin^KO^p53^KO^ (Fig. S3a, phospho-p53, *b* vs. *a* vs. *c*), suggesting that the lack of fortilin increases not only total p53 protein but also phosphorylated and activated p53 protein.

Because fortilin binds p53 and facilitates its proteasome-mediated degradation at the protein level [13], it is expected the *protein* levels of total p53 to be significantly higher in the heart of fortilin^KO^p53^WT^ mice than in those of fortilin^WT^p53^WT^ mice as we observed above. However, this process does not explain why the *message* levels of p53 were also significantly higher in the hearts of fortilin^KO^p53^WT^ mice than in those of fortilin^WT^p53^WT^ mice (Fig. S2c, p53, *a* vs. *b*).

Therefore, we tested the hypothesis that fortilin represses the transcription of p53. We first stably transduced H9C2 rat heart myoblast cells with a lentiviral construct containing both the p53 promoter sequence driving the expression of Gaussia luciferase (GLuc) and the secreted alkaline phosphatase (SEAP) gene under the control of the constitutional CMV promoter (H9C2^p53-pomoter-GLuc/SEAP^ cells). We then transiently silenced fortilin by treating the cells with lentiviral vector containing shRNA against fortilin (sh-fortilin). These cells were irradiated with a moderate X-ray dose (8 Gy) and subjected to the GLuc-SEAP assay (Fig. S3b). Treatment with sh-fortilin effectively silenced fortilin in the cell (Fig. S3c), whereas X-ray irradiation nearly doubled the expression of p53 in the cells (Fig. S3d). In this system, we found that the knockdown of fortilin protein activated the p53 promoter with and without X-ray irradiation (Fig. S3e, columns 1 vs. 3; columns 2 vs. 4). Taken together, these data suggest that fortilin is a transcriptional repressor of p53 transcription in addition to facilitating degradation of p53 protein.

We also found that fortilin^KO^p53^KO^ mice, which express less p53 in the heart than do fortilin^KO^p53^WT^ mice (Fig. 3a and b), survived significantly longer that fortilin^KO^p53^WT^ mice (Fig. 3c, median survival, fortilin^KO^p53^WT^ vs. fortilin^KO^p53^KO^ = 8.0 vs. 18.0 weeks, *P* < 0.0003 by Log-rank Mantel–Cox test). Echocardiography showed that the hearts of fortilin^KO^p53^KO^ mice had better overall heart function (Fig. 3d), as evidenced by greater LVEF and FS, less LV dilatation, and less LV wall thinning (Figs. 3e and S2d). The expression of the αMHC-Cre transgene in the heart did not impact LV systolic function as measured by LVEF and FS (Fig. S2e).

Immunohistochemical staining of myocardium showed that the lack of p53 drastically reduced the expression levels of both BAX and cleaved lamin, which are apoptosis markers (Fig. 3f). Consistently, Western blots revealed that the protein levels of BAX, PUMA, and NOXA were drastically decreased when p53 was deleted from fortilin-deficient hearts (Fig. 3g, *b* vs. *c*). These data suggest that fortilin sustains heart function by negatively regulating p53 and that the lack of fortilin leads to inappropriate overactivation of the p53 pathway, apoptosis and loss of cardiomyocytes, dilated cardiomyopathy, HF, and death.

### Survival benefit via IRE1α inhibition

**Fig. 4:**
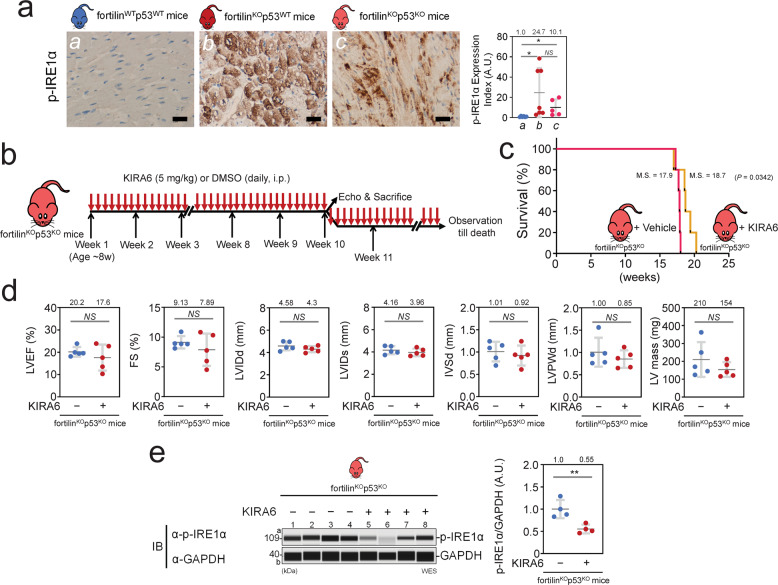
Inhibition of the IRE1α pathway by KIRA6 improved the survival of fortilin^KO^p53^KO^ mice. **a** Activation of the endoplasmic reticulum (ER) stress pathway, as evidenced by phosphorylation of IRE1α, occurred in the hearts of fortilin^KO^p53^WT^ mice. Its activation was not fully reversed by the deletion of p53 in fortilin^KO^p53^KO^ mice. **b** Experimental scheme. Five study and five control mice were weighed, underwent echocardiography, and were sacrificed at week 10 of treatment; the rest of the mice (*N* = 5 each) were observed until their deaths. **c** KIRA6 treatment allowed fortilin^KO^p53^KO^ mice to live significantly longer than did vehicle treatment. **d** Echocardiography showed no significant difference between fortilin^KO^p53^KO^ mice treated with either KIRA6 or vehicle. **e** KIRA6 treatment led to less phosphorylation and activation of IRE1α compared with vehicle treatment. *a* fortilin^WT^p53^WT^ mice (αMHC-Cre^−/−^fortilin^flox/flox^p53^flox/flox^ mice), *b* fortilin^KO^p53^WT^ mice (αMHC-Cre^+/+^fortilin^flox/flox^p53^WT/WT^ mice), *c* fortilin^KO^p53^KO^ mice (αMHC-Cre^+/+^fortilin^flox/flox^p53^flox/flox^ mice), p-IRE1α phosphorylated inositol-requiring enzyme 1 alpha, A.U. arbitrary unit, KIRA6 IRE1α Kinase Inhibiting RNase Attenuator 6, DMSO dimethyl sulfoxide used as vehicle, i.p. intraperitoneal injection, M.S. median survival, LVEF left ventricular (LV) ejection fraction, FS fractional shortening, LVIDd LV internal diameters in diastole, LVIDs LV internal diameters in systole, IVSd interventricular septum thickness in diastole; LVPWd LV posterior wall thickness in diastole, IB immunoblot, α-p-IRE1α anti-phosphorylated IRE1α antibody, GAPDH glyceraldehyde 3-phosphate dehydrogenase, WES an automated capillary-based quantitative Western blot system without total protein evaluation capability; Scale bar = 200 µm; Error bars, means ± SD, statistical analyses performed using Student’s two-sample *t*-test, except for the survival assay, for which the Log-Rank (Mantel–Cox) test was used: NS not statistically significant, *P < 0.05; **P < 0.01 (see also Fig. S4).

Although the deletion of p53 in the heart of fortilin^KO-heart^ mice increased their survival by about 10 weeks (Fig. 3c), it did not fully normalize their survival. Because the ER stress pathway is activated in a failing heart [26] and fortilin binds and negatively regulates IRE1α, a key component of the ER stress pathway [14], we assessed the activation status of IRE1α by immunostaining the heart tissue from fortilin^WT^p53^WT^, fortilin^KO^p53^WT^, and fortilin^KO^p53^KO^ mice (Fig. 4a). We found that the phosphorylated and activated IRE1α (p-IRE1α) levels in the hearts of fortilin^KO^p53^KO^ mice were significantly higher than those of fortilin^WT^p53^WT^ mice, suggesting that even in the absence of p53, the lack of fortilin leads to the activation of the ER stress pathway (Fig. 4a, *a* vs. *c*).

To test the hypothesis that the lack of fortilin in the heart overactivates not only the p53 pathway but also IRE1α and that blockage of both p53 and ER stress pathways is required to normalize the survival of fortilin^KO-heart^ mice, we treated fortilin^KO^p53^KO^ mice with either KIRA6, a selective IRE1α inhibitor [27], or vehicle (Fig. 4b). We found that KIRA6 treatment allowed fortilin^KO^p53^KO^ mice to live modestly longer compared to vehicle treatment (Fig. 4c, median survival of vehicle vs. KIRA6 = 17.86 vs. 18.71 weeks, *P* = 0.0342 by Log-rank Mantel-Cox test). At week 10 of treatment (about 18 weeks of age), both body weights and HW/BW ratios of KIRA6-treated fortilin^KO^p53^KO^ mice were modestly but significantly greater than those of vehicle-treated fortilin^KO^p53^KO^ mice (Fig. S4a and S4b), although echocardiography showed no significant difference between the two groups (Fig. 4d). The hearts of KIRA6-treated fortilin^KO^p53^KO^ mice exhibited less p-IRE1α than those of vehicle-treated fortilin^KO^p53^KO^ mice (Fig. 4e), suggesting that KIRA6 treatment effectively blocked the activation of IRE1α.

## Discussion

Fortilin is abundant in the heart but its function has been unknown. Herein, we have demonstrated that fortilin sustains cardiomyocyte survival at least partly through inhibition of p53 and p53-dependent apoptosis. We found that the lack of fortilin in the heart led to a drastic increase in apoptotic cardiomyocytes, severely decreased heart function, and death and that deletion of p53 from cardiomyocytes nearly totally inhibited cardiomyocyte apoptosis and improved heart function and survival.

The activation of p53 plays a pivotal role in the regulation of cardiac tissue homeostasis under normal conditions [15] and the development of cardiomyocyte damage and HF under biomechanical stress [17, 28, 29]. Transaortic constriction (TAC) and resultant LV pressure overload lead to marked upregulation of P53, induction of BAX, and HF [17]. PUMA is a pro-apoptosis protein induced by P53, and Mandle et al. showed that its deletion from the heart protects mice against TAC-induced HF [28]. The p53 activator quinacrine accelerated TAC-induced HF [29], whereas global p53-deficient mice showed less HF than their wild-type control after TAC [17]. Despite the well-documented role of p53 in the heart, how p53 itself is regulated in the stressed heart has been unclear. Our current work presents evidence that fortilin is a critical negative regulator of p53 in the heart.

We now know that fortilin negatively regulates p53 in three distinct ways. First, fortilin binds to the sequence-specific DNA-binding domain of p53 and prevents it from transcriptionally activating p53-target genes [12]. Second, fortilin promotes the degradation of p53 by inhibiting mouse double minute 2 (MDM2) auto-ubiquitination and promoting MDM2-mediate ubiquitination and degradation of p53 [13]. Finally, fortilin represses the transcriptional activation of the p53 gene itself, as shown by the significantly higher p53 levels in fortilin^KO^p53^WT^ mice compared to fortilin^WT^p53^WT^ mice at the message (Fig. S2c, p53, *a* vs. *b*) level and by activation of the p53 promoter by knockdown of fortilin in the cardiomyocyte cell line (Fig. S3e).

KIRA6, a small molecular weight inhibitor of IRE1α [27], significantly and modestly prolonged the lifespan of fortilin^KO^p53^KO^ mice (Fig. 4c). Although this result may be due to incomplete inhibition of IRE1α by KIRA6 (Fig. 4e), another possibility is that fortilin negatively regulates not only p53 and IRE1α but also unknown apoptosis pathways. This possibility requires further investigation.

There were p53 signals detectable in the Western blot of the total heart lysates from fortilin^KO^p53^KO^ mice (Fig. 3a, α-p53, *c*). Because there are more endothelial cells and fibroblasts combined than cardiomyocytes in the murine hearts [30–32], it is possible that the p53 signals are from those non-cardiomyocyte cells. It is also possible that the Cre-transgene expressed under the αMHC promoter (Fig. S1a-2) was not capable of cleaving the floxed p53 gene in all cardiomyocytes of the fortilin^KO^p53^KO^ heart. To evaluate the status of activated and functional p53 in the heart, we stained the tissue with anti-phospho-p53 antibody. We found that phosphorylated p53 were significantly less in fortilin^KO^p53^KO^ mice than that in fortilin^WT^p53^WT^ mice, suggesting that despite the similar total p53 signals between fortilin^KO^p53^KO^ and fortilin^WT^p53^WT^ hearts by both immunohistochemistry (Fig. S3a, p53, *a* vs. *c*) and Western blots (Fig. 3a, α-p53, *a* vs. *c*), functional p53 is significantly less in fortilin^KO^p53^KO^ hearts than in fortilin^WT^p53^WT^ hearts.

Because cardiomyocytes are not capable of proliferating in normal conditions, their continuous apoptotic loss leads to irreversible progression of HF. Protecting cardiomyocytes against apoptosis represents a new approach in HF gene therapy, distinct from the ones focusing on calcium metabolism and β-adrenergic receptor signal transduction [33]. The current work highlights fortilin as a viable molecular target of HF gene replenishment therapy and prepares us for further investigation of how fortilin expression is maintained in NFHs.

## Methods

### Reagents and materials

Kinase-Inhibiting RNase Attenuator 6 (KIRA6) [27], an inhibitor of IRE1α kinase, was obtained from EMB Millipore (Catalog #: 532281, Burlington, MA). 2,2,2-trichloroethanol (TCE) was purchased from Sigma (St. Louis, MO). Lentiviral particles that contained short-hairpin RNA (shRNA) against human fortilin (lentivirus^sh-fortilin^) and random control sequence (lentivirus^sh-control^) were purchased from Sigma (MISSION^®^ shRNA lentiviral transduction particles). The lentivirus^sh-fortilin^ was experimentally shown to silence rat fortilin (Fig. S3c). Lentiviral particles that contained (a) the murine p53 promoter sequence fused to the GLuc cDNA, (b) the SEAP gene under the constitutional SV40 promoter, and (c) the puromycin resistance gene were obtained from GeneCopoeia (Rockville, MD) (Catalog #: LPP-MPRM33498-LvPG04-200; Lentivirus^p53-promoter-GLuc/SEAP^). The actual promoter sequence is as follows: GGATCTGTGGCTAGCTGGGGTTGGTCATCACCACCGCATGGCGGAGGCACCGGTTCAAAGTCTGTATTTTTCTCCGCTGGGGAACCTTGGGGTACCGGAGCTGGGGCCAGGTCAGGAGGGAGGCTATCCGGAGCTAAGAGTCGCTCCTCCGACGTCTTCATTCTGTAGAGTAAGCCCCCGGAAGGCAGAGGTCGGGCAAGTCTCGCTGAGCCGGCTACCAGCTGCCGAGGCTAGAGTGCATTACCGTTCCCAGGGATGCTCAGAGACCGGAGTCCGCTTTCCTCTTCCGGAAAATGTAAGCCGAACCTAAAGCAATCACCAGGGAACGAGTGTCCAAAGCCAAGCGCCTAGGGTCGCTAGGCGCCGCCAGGGCTTCTTGCTCTCGCGGGAGTCGGGCCACCTTCCGATAGGCTCTCCGCATCCTCCTCCGATTCCGAGCGGGAAGGCGGGAAGGAACGACTTTGCCTACACCTCAAGCGCTGGAGAATTCCTAGAGGTTTCTGGGAGTTGTAGTCTGAACTCTGGGCCTTGGCGAAAACTACACGAGCGCCCCCTACCGTCCCCTGGGGGTAATTCTTAAAGCGCCTATCCTCCCTGGCCTGCAGAGGGCGCATAATTTCTACAGTTTTTGCCCCTCTTGACTATCTTGTTTTGAATCCC

GTAACCTCAGGTTTCCTTTCTCCCCATCTCTCCCCCCTTCTTGTTCCTCTCTTTCCCTTTCTCCCCCGCCCTCCCTTCATTCATTCGACATTTATTATCAAGTTCTTACTGCCTAACCCAGGACTATACAAGGCATTGGGAAAAAAATAGCAATGTTTTCTAGTTCTTAATCTCCATAAAGTTTTCGTTGCTGTGCAATTAAAGGCTGTGAAAACAGTCTTTACAGAGAGTGATAAGGACTGTACAGGAAATTAAACACGGTGGTGCGATACCAAGTATCTCGGAGAACACGTTAGATTGAGATACTATGAAAAGCCTTTCTAAAGTGACATTTTAGCTAATGAGGGGAAAAAGAACTTAGGGGCCCGTGTTGGTTCATCCCTGTACTTGGAAGGCCTAAAGCAGGAAGACGGCCGCGAATTCCAGGCCAGCCTTGGCTACAAAGACTCTGTCTTAAAAATCCAAAAAGATGGCTATGACTATCTAGCTGGATAGGAAAGAGCACAGAGCTCAGAACAGTGGCGGTCCACTTACGATAAAAACTTAATTCTTTCCACTCTTTATACTTGACACAGAGGCAGGAGTCCTCCGAATCGGTTTCCACCCATTTTGCCCTCACAGCTCTATATCTTAGACGACTTTTCACAAAGCGTTCCTGCTGAGGGCAACATCTCAGGGAGAATCCTGACTCTGCAAGTCCCCGCCTCCATTTCTTGCCCTCAACCCACGGAAGGACTTGCCCTTACTTGTTATGGCGACTATCCAGCTTTGTGCCAGGAGTCTCGCGGGGGTTGCTGGGATTGGGACTTTCCCCTCCCACGTGCTCACCCTGGCTAAAGTTCTGTAGCTTCAGTTCATTGGGACCATCCTGGCTGTAGGTAGCGACTACAGTTAGGGGGCAC.

### Cell culture

MycoFluor™ (ThermoFisher Scientific-Molecular Probe, Eugene, OR) was used to detect mycoplasma contamination when appropriate. The H9C2(2-1) (ATCC^®^ CRL-1446™) cell line (H9C2 hereafter) was directly obtained from American Type Culture Collection (Manassas, VA, USA). Cells were maintained in Dulbecco’s modified Eagle’s medium (Corning, Corning, NY, Catalog #: 0-013-CV) with 10% fetal bovine serum (FBS) (Catalog #: 10082-147; ThermoFisher Science–Gibco, Waltham, MA) at 37 °C in an atmosphere containing 5% CO_2_. H9C2 cells stably harboring the lentiviral construct that contained both the p53 promoter sequence driving the expression of GLuc and the SEAP gene under the control of the constitutional SV40 promoter were generated by transducing the cells with Lentivirus^p53-promoter-GLuc/SEAP^ and selecting them with puromycin (H9C2^p53-promoter-GLuc/SEAP^). They were maintained in puromycin-containing (0.3 µg/mL) media.

### Dual-luciferase assay

On the first day, H9C2^p53-promoter-GLuc/SEAP^ cells were transiently transduced by either (lentivirus^sh-fortilin^) or (lentivirus^sh-control^) at the multiplicity of infection (MOI) of 1. The next day, cells were irradiated using the RS-2000 X-ray irradiator (QuaStar, Buford, GA) at 8 Gy. Seventy-two hours after the irradiation, conditioned media were collected and subjected to both SEAP and luciferase assays using the Secrete-Pair Dual Luminescence Assay Kit according to the manufacturer’s instruction (GeneCopoeia). The degree of activation of the p53 promoter was assessed by dividing the luciferase activity units by the SEAP activity units and expressing it in arbitrary units (A.U.).

### General veterinary care

Mice were given standard mouse chow (Lab Diet; Catalog #: 5L0D) and water ad libitum, maintained on a 12-h light–dark cycle and seen by a scientist daily to observe their behavior and health. Any animal that displayed substantive signs of distress anytime during the protocol, including but not limited to poor grooming, hunched back posture, or a loss in weight exceeding 10% of the weights at the initiation of the experiments, were identified and treated accordingly, including removal from the experiment and euthanasia. For mouse experiments where grouping was based on pharmacological treatments, mice of the same genotype were randomly assigned to treatment and control groups.

### Generation of heart-specific fortilin KO (fortilin^KO-heart^) mice

Fortilin^flox/flox^ mice, in which the fortilin gene was flanked by the LoxP sequence to allow tissue-specific deletion, were generated using the standard homologous recombination technique described previously: we micro-injected a mutant C57BL6 embryonic stem cell (ESC) line that contained two LoxP sequences flanking all six fortilin exons into C57BL6 blastocysts [14]. The resultant fortilin^flox/flox^ mice were fully in the C57BL/6J genetic background from the beginning. Fortilin^flox/flox^ mice were then crossed with a transgenic flipase strain to remove the neomycin resistance gene cassette. To generate fortilin^KO-heart^ mice, fortilin^floxflox^ mice were crossed with C57BL/6J mice overexpressing the Cre transgene under the control of cardiac-specific α-myosin heavy chain (Myh6) promoter [22] (αMHC-Cre^+/+^, Jackson Laboratory, Ann Arbor, ME, Stock # 011038, already backcrossed to C57BL/6J for eight generations by the donating laboratory). Genotyping of fortilin^KO-heart^ and fortilin^WT-heart^ mice was performed on tail-derived genomic DNA using standard PCR-based methods. The presence of the αMHC-Cre transgene was detected using the following primer sets: 5′-ATGACAGAC AGATCCCTCCTATCTCC-3′ (M1) and 5′-CTCATCACTCGTTGCATCATCGAC-3′ (M2). αMHC-Cre^+/−^ mice yielded a 300 bp amplified fragment and αMHC-Cre^−/−^ mice yielded no amplicons. To determine the hemi- (αMHC-Cre^+/−^) or homo- (αMHC-Cre^+/+^) zygosity of the MHC-Cre transgene, RT-qPCR was performed using the primers and probe sets described previously [14] (see below). We used the 2^–ΔΔCT^ method [34] to determine the expression levels of the αMHC-Cre transgene relative to the internal control levels in the sample, according to the Protocol 20627 of the Jackson Laboratory.

- αMHC-Cre-transgene:
  - Forward primer (oIMR1084): 5’-GCGGTCTGGCAGTAAAAACTATC-3’
  - Reverse primer (oIMR1085): 5’-GTGAAACAGCATTGCTGTCACTT-3’
  - Probe (13593): 5’-FAM-AAACATGCTTCATCGTCGGTCCGG-IBFQ-3’
  - FAM, 6-carboyfluorescein; IBFQ, Iowa Black™ double-quenched probe (Integrated Data Technologies (IDT), Coralville, IA)
- Internal control to calculate ΔCT for the αMHC-Cre-transgene:
  - Forward primer (oIMR1544): 5’-CACGTGGGCTCCAGCATT-3’
  - Reverse primer (oIMR3580): 5’-TCACCAGTCATTTCTGCCTTTG-3’
  - Probe (TmoIMR0105): 5’-JOE-CCAATGGTCGGGCACTGCTCAA-IBFQ-3’
  - The target gene is *Apob* (apolipoprotein B) [35].

The presence of the LoxP-fortilin-LoxP knock-in construct was verified using the following primer sets: 5′-TGGACCCTGACTTTCATCACCTC-3′ (F1) and 5′- GTCATCTAACCTTACCCCAGTAAGC-3′ (F2): fortilin^flox/flox^ mice yielded a 405 bp fragment and wild-type fortilin mice (fortilin^WT/WT^ mice) yielded a 280 bp fragment.

### Generation of heart-specific fortilin p53 double KO (fortilin^KO^p53^KO^) mice

To generate a strain of C57BL/6J mice that lack both fortilin and p53 in the heart, fortilin^KO-heart^ mice were crossed with p53^flox/flox^ mice available from the Jackson Laboratory (Stock Number: 008462, p53^LoxP^). The status of the αMHC-Cre-transgene was evaluated by both PCR and RT-qPCR as described above, and the status of fortilin and p53 loci was determined using the following PCR-based genotyping strategies:

- Fortilin locus:
  - Forward primer: 5’-TGGACCCTGACTTTCATCACCTC-3’
  - Reverse primer: 5’-GTCATCTAACCTTACCCCAGTAAGC-3’
  - Expected size of the mutant (floxed fortilin gene) allele: 405 bp
  - Expected size of the wildp-type allele: 280 bp
- P53 locus:
  - Forward primer: 5’-GGTTAAACCCAGCTTGACCA-3’
  - Reverse primer: 5’-GGAGGCAGAGACAGTTGGAG-3’
  - Expected size of the mutant (floxed fortilin gene) allele: 390 bp
  - Expected size of the wild-type allele: 270 bp

### KIRA6 rescue experiment

For the KIRA6 rescue experiment, 8-week-old male fortilin^KO^p53^KO^ mice were daily and intraperitoneally injected with either 5 mg/kg of KIRA6 in solution (3% ethanol, 7% Tween-80, and 90% saline) or the same solution without KIRA6 as vehicle (a) for the subsequent 10 weeks for the sacrifice group (*N* = 5 each) or (b) until death for the survival analysis group (*N* = 5 each). Mice were examined daily for their behavior and health. Mice in the sacrifice group were weighed and underwent transthoracic echocardiography before they were sacrificed, and their hearts were harvested and processed as described elsewhere in “Methods” section.

### Mouse echocardiography

Mouse transthoracic echocardiography was performed using a Vevo 2100 High-Resolution Imaging System (FUJIFILM VisualSonics, Toronto, ON, Canada) on mice anesthetized initially by 4% isoflurane in 1 L/min of O_2_ and then by 0.5–3.0% isoflurane and placed on a heated platform, as described previously [36]. We imaged the heart in the parasternal long-axis view in both 2D and M-modes. We obtained the following parameters using the software on the system: left ventricular ejection fraction (LVEF, %), fractional shortening (FS, %), left ventricular internal diameter in systole (LVIDs, mm), left ventricular internal diameter in end-diastole (LVIDd, mm), interventricular septum thickness in end diastole (IVSd, mm), left ventricular posterior wall thickness in end diastole (LVPWd, mm), and LV mass (mg). For the initial characterization of fortilin^KO-heart^ mice, the baseline echo was obtained when they were 7 weeks of age.

### Processing of the mouse hearts

After being weighed, mice were sacrificed by isoflurane inhalation until effective, followed by exsanguination. Both the heart and lungs were routinely harvested. The liver, kidney, and spleen were harvested, when appropriate, for protein and RNA analyses. The harvested hearts and lungs were rinsed with phosphate-buffered saline (PBS), drained on absorbent paper, and weighed. The ratio of heart weight to body weight (HW/BW ratio) as well as that of lung weight to body weight (LW/BW ratio) was then calculated. For the sagittal cross-sectional analysis of the heart, the entire heart was fixed in 4% paraformaldehyde and subjected to paraffin embedding, sectioning, and hematoxylin and eosin staining according to the standard protocol. The right ventricle and atria were removed from the left ventricle (LV). The LV was then divided into three sections of equal size (basal, mid-ventricular, and apical). The mid-ventricular part of the LV was fixed in 4% paraformaldehyde before being embedded in paraffin for immunohistochemistry. The apical portion of the LV was flash-frozen for subsequent protein extraction, and the basal portion of the LV was placed directly into Tri-Reagent (Molecular Research Center, Cincinnati, OH, USA) and frozen for subsequent RNA extraction.

### RT-qPCR

RT-qPCR was performed as described previously [14]. We used the 2^–ΔΔCT^ method [34] to calculate the expression levels of a gene in question relative to the 18S rRNA levels in the sample. Briefly, the hearts (and, when appropriate, other organs) of mice were harvested into Tri-Reagent. RNA was isolated following the manufacturer’s instructions and treated with DNAse (ABI, Foster City, CA, USA). RT-qPCR was performed in quadruplicate with exactly 50 ng of total RNA using the TaqMan^®^ RT-PCR kit (Applied Biosystems [ABI] at Life Technologies, Grant Island, NY, USA) in the ABI Step One Plus Real-Time PCR system and the following primer and probe sets (Integrated DNA Technologies):

1. Mouse *Fortilin*:
  - Forward primer: 5′-TCCGACATCTACAAGATCCGG-3′,
  - Reverse primer: 5′-ATCTTGCCCTCCACCTCCA-3′,
  - Probe: 5′-FAM-AGATCGCGG/ZEN/ACGGGCTGTGC-IBFQ-3′
  - ZEN™ = an internal quencher to enhance the quenching activity of the 3’ quencher IBFQ (IDT)
2. Mouse *Col1*:
  - Forward primer: 5′-GAAACCCGAGGTATGCTTGA-3′,
  - Reverse primer: 5′-GTTGGGACAGTCCAGTTCTT-3′,
  - Probe: 5′-FAM-TGTGCGATGACGTGCAATGCAATG-IBFQ-3′
3. Mouse *Myh7*
  - Forward primer: 5′-CCATCTCTGACAACGCCTATC-3′,
  - Reverse primer: 5′-GGATGACCCTCTTAGTGTTGAC-3′,
  - Probe: 5′-FAM-TCAGTCCATCCTCATCACCGGAGA-IBFQ-3′
4. Mouse *ANF*
  - Forward primer: 5′-TCCGATAGATCTGCCCTCTT-3′,
  - Reverse primer: 5′-CTCCAATCCTGTCAATCCTACC-3′,
  - Probe: 5′-FAM-AAAGCAAAC/ZEN/TGAGGGCTCTGCTCG-IBFQ-3′
5. Mouse *Acta1*
  - Forward primer: 5′-CTCCCTGGAGAAGAGCTATGA-3′,
  - Reverse primer: 5′-CGATAAAGGAAGGCTGGAAGAG-3′,
  - Probe: 5′-FAM-CATCGGCAATGAGCGTTTCCGTTG-IBFQ-3′
6. Mouse *Serca2*
  - Forward primer: 5′-CATCAGTATGACGGGCTTGTAG-3′,
  - Reverse primer: 5′-CTCGGTAGCTTCTCCAACTTTC-3′,
  - Probe: 5′-FAM-AGCCACCATCTGTGCTCTGTGTAA-IBFQ-3′
7. Mouse *p53*
  - Forward primer: 5′-CAGCTTTGAGGTTCGTGTTTG-3′,
  - Reverse primer: 5′-AGTTCAGGGCAAAGGACTTC-3′,
  - Probe: 5′-FAM-TCTTCTTCT/ZEN/GTACGGCGGTCTCTCC-IBFQ-3′
8. Mouse *Bax*
  - Forward primer: 5′-TTGCTGATGGCAACTTCAACTGGG-3′,
  - Reverse primer: 5′-TGTCCAGCCCATGATGGTTCTGAT-3′,
  - Probe: 5′- FAM-TTTGCTAGC/ZEN/AAACTGGTGCTCAAGGC-IBFQ-3′
9. Mouse *Puma*
  - Forward primer: 5′-ATGGCGGACGACCTCAAC-3′,
  - Reverse primer: 5′-AGTCCCATGAAGAGATTGTACATGAC-3′,
  - Probe: 5′-FAM-AGCAGCATC/ZEN/GACACCGACCCTCAC-IBFQ-3′
10. Mouse *Noxa*
  - Forward primer: 5′-TGCACCGGACATAACTGTGGTTCT-3′,
  - Reverse primer: 5′-TGAGCACACTCGTCCTTCAAGTCT-3′,
  - Probe: 5′-FAM-AAAGAGCAG/ZEN/GATGAGGAGCCCAAGC-IBFQ-3′
11. Mouse *18S rRNA*
  - Forward primer: 5′- GCCGCTAGAGGTGAAATTCT-3′,
  - Reverse primer: 5′-TCGGAACTACGACGGTATCT-3′,
  - Probe: 5′-JOEN-ACCAGAGCG/ZEN/AAA GCATTTGCCAAG-IBFQ-3′
  - JOEN = 6-carboxy-4′,5′-dichloro-2′,7′-dimethoxyfluorescein

### Western blot analyses

SDS–PAGE and Western blot analyses were performed as described previously [8, 37–40] on the lysates from the mouse organs. The following primary antibodies were used at the indicated dilutions/concentrations:

- Anti-fortilin (Abnova, Taipei City, Taiwan; Clone 2C4, H00007178-M03; 1:1000 dilution)
- Anti-p53 (Santa Cruz Biotechnology, Dallas, TX; sc-6243; 1:1000 dilution)
- Anti-GAPDH (Santa Cruz Biotechnology; Clone 6C5, sc-32233; 1:10,000 dilution)
- Anti-Bax (Santa Cruz Biotechnology; sc-493; 1:1000 dilution)
- Anti-PUMA (Cell Signaling Technology, Danvers, MA; 7467; 1:1000 dilution)
- Anti-NOXA (EMD Millipore, Billerica, MA; AB5761; 1:1000 dilution)
- Anti-Tropomyosin (Santa Cruz Biotechnology; sc-28543; 1:1000 dilution)

All antibodies were used with appropriate IRDye680LT- or IRDye800CW-conjugated secondary antibodies (LI-COR, Lincoln, NE, USA). The signal intensities of protein bands were quantified using the Odyssey Infrared Imaging System (LI-COR) and normalized to the signal intensity of the loading control protein (GAPDH or tropomyosin) and expressed in A.U. To quantify p53 expression in response to X-ray irradiation, 0.5% (v/v) TCE was added to a polyacrylamide gel before polymerization. After standard SDS–PAGE, the gel was UV-irradiated on the Bio-Rad ChemiDoc MP Imaging System (Bio-Rad, Hercules, CA) for 2 min. The image was electronically captured, and the cumulative band densities were calculated to assess loading conditions as previously described [41]. The signal intensity of Western blot bands was divided by that of the TCE bands to derive the p53 expression index. Results were expressed in A.U.

### JESS/WES analysis

To evaluate the expression levels of fortilin in human hearts, we obtained de-identified tissue lysates of human hearts from patients with NFHs, non-ischemic cardiomyopathy (NICM), and ischemic cardiomyopathy (ICM) from the Duke Human Heart Repository (Duke University, Durham, NC). An automated capillary-based quantitative Western blot system called JESS™ (Protein Simple, San Jose, CA) was used to (a) detect fortilin (anti-fortilin antibody, MBL International Corporation, Woburn, MA; PM017; 1:10 dilution) and (b) visualize total proteins loaded as described previously [19, 20]. Compass Software (v3.1, Protein Simple) was used to calculate a fortilin expression index by dividing the area under the curve of a fortilin peak by the total proteins loaded in the same capillary (“in-capillary normalization”). The fortilin expression index was expressed in A.U.

To quantify phosphorylated IRE1α, the mouse heart lysates from fortilin^KO^p53^KO^ mice that were treated with either vehicle or KIRA6 were subjected to WES™ (Protein Simple), an automated capillary-based quantitative Western blot system, to detect p-IRE1α (anti-phospho-IRE1α antibody, Novus Biologicals, Centennial, CO; NB100-2323; 1:10 dilution) and glyceraldehyde 3-phosphate dehydrogenase (GAPDH; anti-GAPDH antibody; Santa Cruz Biotechnology; Clone 6C5, sc-32233; 1:250 dilution); the latter served as the loading control. JESS™, but not WES™, can visualize the total proteins loaded for normalization. Compass Software (v3.1) was used to calculate a fortilin expression index by dividing the area under the curve of a fortilin peak by that of a GAPDH peak loaded in the same capillary (“in-capillary normalization”). The fortilin expression index was expressed in A.U.

### Immunohistochemistry of mouse hearts

Mouse hearts were fixed in 4% paraformaldehyde and embedded in paraffin before they were sectioned at 5 µm thickness. Immunohistochemistry of mouse hearts was performed as described previously [42] using the primary antibodies listed below and 3,3’-diaminobenzidine (DAB) as the chromogen. Myocardial fibrosis was quantified by both Masson staining and picrosirius red staining as previously described [43, 44]. All immunostained sections were digitally imaged using the EasyScan Digital Slide Scanner (Motic, San Francisco, CA). Using the ImageJ software (National Institutes of Health, Bethesda, MD), expression indices were calculated by dividing the DAB-positive area (or signal-positive area for Masson and Picrosirius staining) by the region of interest (ROI), and results were expressed in A.U. For quantification of phospho-p53 positive cells in the heart, DAB-positive nuclei were counted in a randomly selected field on the DAB-only images generated by the color deconvolution function of ImageJ (version 1.53k; National Institutes of Health, Bethesda, MD). The process was repeated for 5 different fields per mouse for 5 mice.

- Cleaved lamin A (Cell Signaling Technology; 2035; 1:100 dilution). The cleavage of lamin is a well-characterized event in apoptosis [45].
- BAX (Santa Cruz Biotechnology; sc-493; 1:500 dilution)
- p-IRE1α (Abcam, Cambridge, MA; ab48187; 1:4000 dilution)
- p53 (Novocastra, Wetzlar, Germany; NCL-L-p53-CM5p; 1:250 dilution)
- phosphorylated p53 (phospho-p53, Cell Signaling Technologies, Danvers, MA; #9284; 1:100 dilution)

### TUNEL staining

Terminal deoxynucleotidyl transferase dUTP nick end labeling (TUNEL) staining of heart tissue was performed on formalin-fixed, paraffin-embedded samples as previously described [12, 46] using the FragEL^TM^ DNA Fragmentation Detection Kit (EMD Millipore-Calbiochem) following the manufacturer’s instructions. TUNEL-positive cells within about 0.145 mm^2^ of the ROI were counted, and TUNEL indices were calculated as the number of TUNEL-positive cells per unit area (in mm^2^). Results are expressed in A.U.

### Cardiomyocyte area

To assess the presence of cardiomyocyte hypertrophy, the heart tissue that had been formalin-fixed and paraffin-embedded was sectioned at 5 µm thickness and stained with laminin (ThermoScientific, Waltham, MA; RB082A; 1:100 dilution). After stained sections were digitally captured using the EasyScan Digital Slide Scanner, cardiomyocyte areas were measured using ImageJ and expressed in A.U. as described previously [47]. At least five distinct areas were quantified per mouse sample.

### Ethics statement

This study was performed in accordance with the recommendations in the Guide for the Care and Use of Laboratory Animals of the National Institutes of Health. All experiments involving animals were approved by the Institutional Animal Care and Use Committees of our institution. Human tissue samples used for this study were procured from the Duke Human Heart Repository (DHHR), which is a Duke University Health System Institutional Review Board (DUHS IRB) approved tissue repository. Samples were procured by the DHHR using written informed consent or a waiver of consent for discarded tissues. No HIPAA information was provided with any of the samples used in this study. Human myocardium was acquired from the left ventricular free wall of explanted hearts following cardiac transplantation. Non-failing (NF) left ventricular tissue was acquired from donors whose hearts were not utilized for transplant, thus becoming available for research.

### Statistical analysis

All measurements were taken from distinct samples (biological replicates) and the size of biological samples (*N*) is indicated in either figures or the main text. The degree of the spread of data was expressed by the standard deviation (±SD). The difference between the control and study groups was analyzed using unpaired two-tailed Student’s *t*-test for two groups or one-way analysis of variance (ANOVA) followed by Fisher’s pairwise multiple comparisons for multiple groups. *P* < 0.05 was considered to be statistically significant. For survival analyses, Kaplan–Meier survival curves were generated, and the Log-Rank (Mantel–Cox) test was used to compare the curves. The numbers of mice used in in vivo experiments were determined by (i) power analysis, assuming an *α* error rate of 0.05, a *β* error rate of 0.20, and an expected difference of 25%, in Minitab 17 (State College, PA) or (ii) our previous dataset and experience from similar experiments performed as part of past research. A similar variance was observed between the groups that were statistically compared. No data were excluded unless outliers were identified and verified by the outlier tests (Minitab 17). Although the scientists were not blinded to allocation during experiments and readouts evaluation, all readouts from the experiments were predetermined, highly objective, and obtained according to the validated protocols.

## Supplementary information

SUPPLEMENTAL MATERIAL

## Data Availability

The authors declare that the data supporting the findings of this study are available within the paper and its supplementary information files. All relevant data are available from the authors upon request.
