# Supplementary material for: Fortilin inhibits p53, halts cardiomyocyte apoptosis, and protects the heart against heart failure
Source: Cell Death Discov. 2021 Oct 23;7:310. doi: 10.1038/s41420-021-00692-w (PMC8542040; doi:10.1038/s41420-021-00692-w)
Supplement: Supplementary file 1 — SUPPLEMENTAL MATERIAL [file 41420_2021_692_MOESM1_ESM.pdf]

SUPPLEMENTARY MATERIALS

Fortilin inhibits p53, halts cardiomyocyte apoptosis, and protects the heart against heart failure

Authors: Preedakorn Chunhacha, Decha Pinkaew, Patuma Sinthujaroen, Dawn E. Bowles, and Ken Fujise

SUPPLEMENTARY FIGURES & LEGENDS

Fig. S1. **Mice lacking fortilin in the heart died prematurely due to severe heart failure.** Abbreviations: NdeI, restriction enzyme NdeI (CA|TATG); NsiI, restriction enzyme NsiI (ATGCA|T); F1/F2, PCR primer pair to evaluate the presence of the LoxP sequence and conditional allele; M1/M2, PCR primer set to evaluate the presence of the Cre-transgene; αMHC-Cre, Cre-transgene under the control of the alpha myosin heavy chain promoter; A.U., arbitrary units; WT, fortilin$^{WT\text{-}heart}$ (or αMHC-Cre$^{-/-}$fortilin$^{flox/flox}$) mice that express fortilin normally in the heart; KO, fortilin$^{KO\text{-}heart}$ (or αMHC-Cre$^{+/+}$fortilin$^{flox/flox}$) mice that do not express fortilin in the heart; HW/BW ratio, the ratio of heart weight to body weight; LW/BW ratio, the ratio of the lung weight to body weight; Col1, collagen type I alpha 1; Myh7, myosin heavy chain 7; ANF, atrial natriuretic factor; Acta1, alpha-1 skeletal muscle actin; Serca2, sarco-endoplasmic reticulum Ca^{2+}-ATPase; Error bars, means ± SD, statistical analyses performed using Student's two-sample t-test; NS, not statistically significant; *, $P < 0.05$; ***, $P < 0.005$; ****, $P < 0.001$ (*See also Fig. 1*). **(a)** A strain of mice lacking fortilin in the cardiomyocytes (fortilin$^{KO\text{-}heart}$) was generated by crossing fortilin$^{flox/flox}$ mice (1) with mice that harbor the αMHC-Cre transgene (2). The mice expressed no fortilin in cardiomyocytes (3a) while they express fortilin in all other cell types (3b). **(b)** Fortilin message levels were drastically lower in the hearts of fortilin$^{KO\text{-}heart}$ mice than in those of fortilin$^{WT\text{-}heart}$ mice. **(c)** At the 8th week of age, both the HW/BW and LW/BW of fortilin$^{KO\text{-}heart}$ mice were significantly greater than those of fortilin$^{WT\text{-}heart}$ mice. **(d)** RT-qPCR assays reveal increased expression levels of the fetal

heart genes, namely, Col1, Myh7, and ANF in fortilin$^{KO\text{-}heart}$ mice, as compared with those in fortilin$^{WT\text{-}heart}$ mice.

Fig. S2. **The lack of p53 partially rescued the premature death of fortilin$^{KO\text{-}heart}$ mice.** Abbreviations: *a*, fortilin^{WT}p53^{WT} mice (αMHC-Cre$^{-/-}$fortilin$^{flox/flox}$p53$^{flox/flox}$ mice); *b*, fortilin^{KO}p53^{WT} mice (αMHC-Cre$^{+/+}$fortilin$^{flox/flox}$p53$^{WT/WT}$ mice); *c*, fortilin^{KO}p53^{KO} mice (αMHC-Cre$^{+/+}$fortilin$^{flox/flox}$p53$^{flox/flox}$ mice); αMHC-Cre-Tg, Cre-transgene under the control of the alpha myosin heavy chain promoter; A.U., Arbitrary unit; LVEF, left ventricular (LV) ejection fraction; FS, fractional shortening; LVIDd, LV internal diameters in diastole; LVIDs, LV internal diameters in systole; IVSd, interventricular septum thickness in diastole; LVPWd, LV posterior wall thickness in diastole; Error bars, means ± SD, statistical analyses performed using (a) Student's two-sample t-test and (b) ANOVA with Fisher's multiple comparison for the evaluation of two and three experiment groups, respectively; *NS*, not statistically significant; *, $P < 0.05$; **, $P < 0.01$; ****, $P < 0.001$ (*See also Fig. 3*). **(a)** fortilin^{WT}p53^{WT} *(a)*, fortilin^{KO}p53^{WT} *(b)*, and fortilin^{KO}p53^{KO} *(c)* mice were generated by crossing fortilin$^{KO\text{-}heart}$ mice with p53$^{flox/flox}$ mice. **(b)** PCR analyses show the successful generation of fortilin^{WT}p53^{WT}, fortilin^{KO}p53^{WT}, and fortilin^{KO}p53^{KO} mice. **(c)** RT-qPCR showed that the fortilin message levels were significantly lower in fortilin^{KO}p53^{WT} and fortilin^{KO}p53^{KO} mice than in fortilin^{WT}p53^{WT} mice, while p53 message levels were significantly lower in fortilin^{KO}p53^{KO} mice than in fortilin^{WT}p53^{WT} or fortilin^{KO}p53^{WT} mice. **(d)** Echocardiography revealed that the hearts of fortilin^{KO}p53^{KO} mice had better overall heart function than those of fortilin^{KO}p53^{WT} mice, as evidenced by greater FS. **(e)** The expression of the αMHC-Cre

transgene did not impact LV systolic function as measured by LVEF and FS. We compared heart function of 7-week-old fortilin^{WT}p53^{WT} mice (αMHC-Cre$^{-/-}$ fortilin$^{flox/flox}$p53$^{flox/flox}$) with that of 18-week old mice whose hearts expressed the Cre-transgene (N = 6 each). There was no difference in LVEF or FS between these two strains.

Fig. S3. **Fortilin decreases p53 phosphorylation in the heart and is a transcriptional repressor of p53.** Abbreviations: H&E, hematoxylin and eosin; DAB, 3,3'-diaminobenzidine; scale bar = 100 μm; brown arrows, phospho-p53-positive nuclei; GLuc, Gaussia luciferase; SEAP, secreted alkaline phosphatase; H9C2, rat myoblast cell line; sh-control, lentivirus containing the short-hairpin control RNA; sh-fortilin, lentivirus containing the short-hairpin RNA against fortilin; H9C2$^{p53\text{-}promoter\text{-}GLuc/SEAP}$, H9C2 cells stably transduced by the lentiviral vector that contains (i) p53-promoter sequence fused to GLuc cDNA, (ii) SEAP gene under the control of the constitutional SV40 promoter, and (iii) puromycin resistance gene; IB, immunoblot; α-fortilin, anti-fortilin antibody; α-p53, anti-p53 antibody; TCE, 2,2,2-trichloroethanol staining; A.U., Arbitrary unit; Error bars, means ± SD, statistical analyses performed using Student's two sample t-test for the comparison of two samples and ANOVA with Fisher's multiple comparison for four samples; NS, not statistically significant; *, $P < 0.05$; ****, $P < 0.001$ (*See also Fig. 3*). **(a)** p53 immunogenicity in the heart is greater in fortilin^{KO}p53^{WT} than fortilin^{WT}p53^{WT} and fortilin^{KO}p53^{KO} mice. There is no significant difference in p53 immunogenicity between fortilin^{WT}p53^{WT} and fortilin^{KO}p53^{KO} mice. The immunogenicity against phosphorylated p53 (phospho-p53) in the heart is significantly greater in

fortilin^{KO}p53^{WT} mice than in fortilin^{WT}p53^{WT} mice. It is significantly greater in fortilin^{WT}p53^{WT} than in fortilin^{KO}p53^{KO} mice (N = 5 each). **(b)** Experimental scheme of the GLuc-SEAP reporter assay. **(c)** sh-fortilin successfully silenced fortilin in H9C2$^{p53\text{-}promoter\text{-}GLuc/SEAP}$ cells. **(d)** X-ray irradiation (8 Gy) induced p53 in H9C2$^{p53\text{-}promoter\text{-}GLuc/SEAP}$ cells. **(e)** The dual GLUc-SEAP assays (N = 12 each) revealed that fortilin blocked p53 promoter activation both in the presence and absence of X-ray irradiation.

Fig. S4. Inhibition of the IRE1α pathway modestly improved the survival of fortilin^{KO}p53^{KO} mice. Abbreviations: fortilin^{KO}p53^{KO} mice (αMHC-Cre$^{+/+}$fortilin$^{flox/flox}$p53$^{flox/flox}$ mice); A.U., arbitrary unit; KIRA6, IRE1α Kinase Inhibiting RNase Attenuator 6; HW/BW, the ratio of the heart weight to the body weight; Error bars, means ± SD, statistical analyses performed using Student's two-sample t-test; *, $P < 0.05$; ***, $P < 0.005$ (*See also Fig. 4*). **(a)** KIRA6-treated fortilin^{KO}p53^{KO} mice were significantly heavier than vehicle-treated fortilin^{KO}p53^{KO} mice at week 10 of treatment. **(b)** The HW/BW ratio was significantly greater in KIRA-treated fortilin^{KO}p53^{KO} mice than in vehicle treated fortilin^{KO}p53^{KO} mice. **(c)** Fortilin inhibited p53 and, to a lesser degree, IRB1α in the heart, protecting it from BAX/NOXA/PUMA-mediated cardiomyocyte apoptosis and progression of HF.

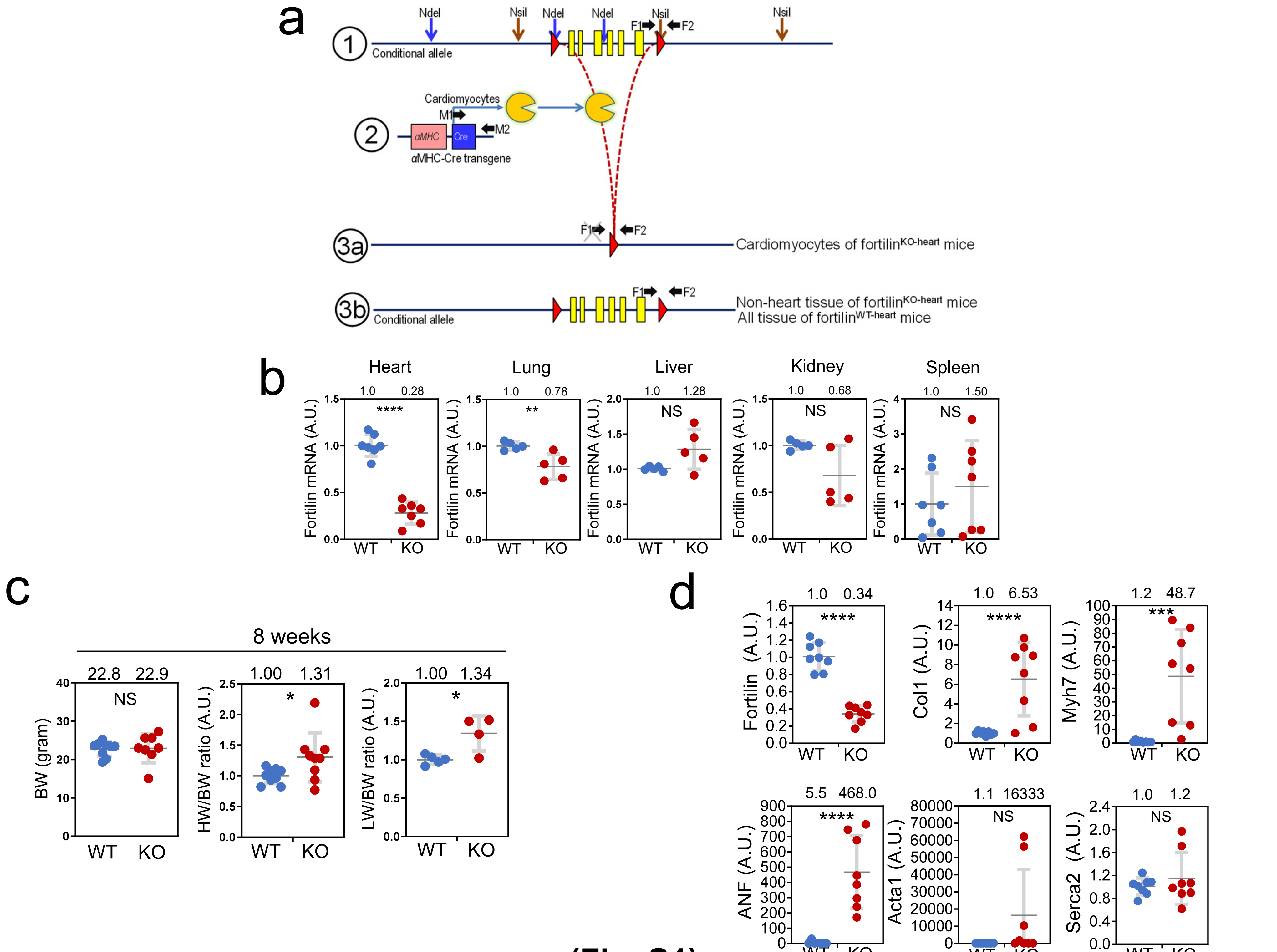
a
NdeI
NsiI
NdeI
NdeI
NsiI
NsiI
F1
F2
1
Conditional allele
Cardiomyocytes
M1
M2
2
αMHC
Cre
αMHC-Cre transgene
3a
Cardiomyocytes of fortilin$^{KO-heart}$ mice
3b
Conditional allele
Non-heart tissue of fortilin$^{KO-heart}$ mice
All tissue of fortilin$^{WT-heart}$ mice
b
Heart
Lung
Liver
Kidney
Spleen
Fortilin mRNA (A.U.)
1.0 0.28
1.0 0.78
1.0 1.28
1.0 0.68
1.0 1.50
****
**
NS
NS
NS
WT KO
c
8 weeks
22.8 22.9
1.00 1.31
1.00 1.34
NS
*
*
BW (gram)
HW/BW ratio (A.U.)
LW/BW ratio (A.U.)
d
1.0 0.34
1.0 6.53
1.2 48.7
5.5 468.0
1.1 16333
1.0 1.2
Fortilin (A.U.)
Col1 (A.U.)
Myh7 (A.U.)
ANF (A.U.)
Acta1 (A.U.)
Serca2 (A.U.)
****
****
***
****
NS
NS

(Fig. S1)

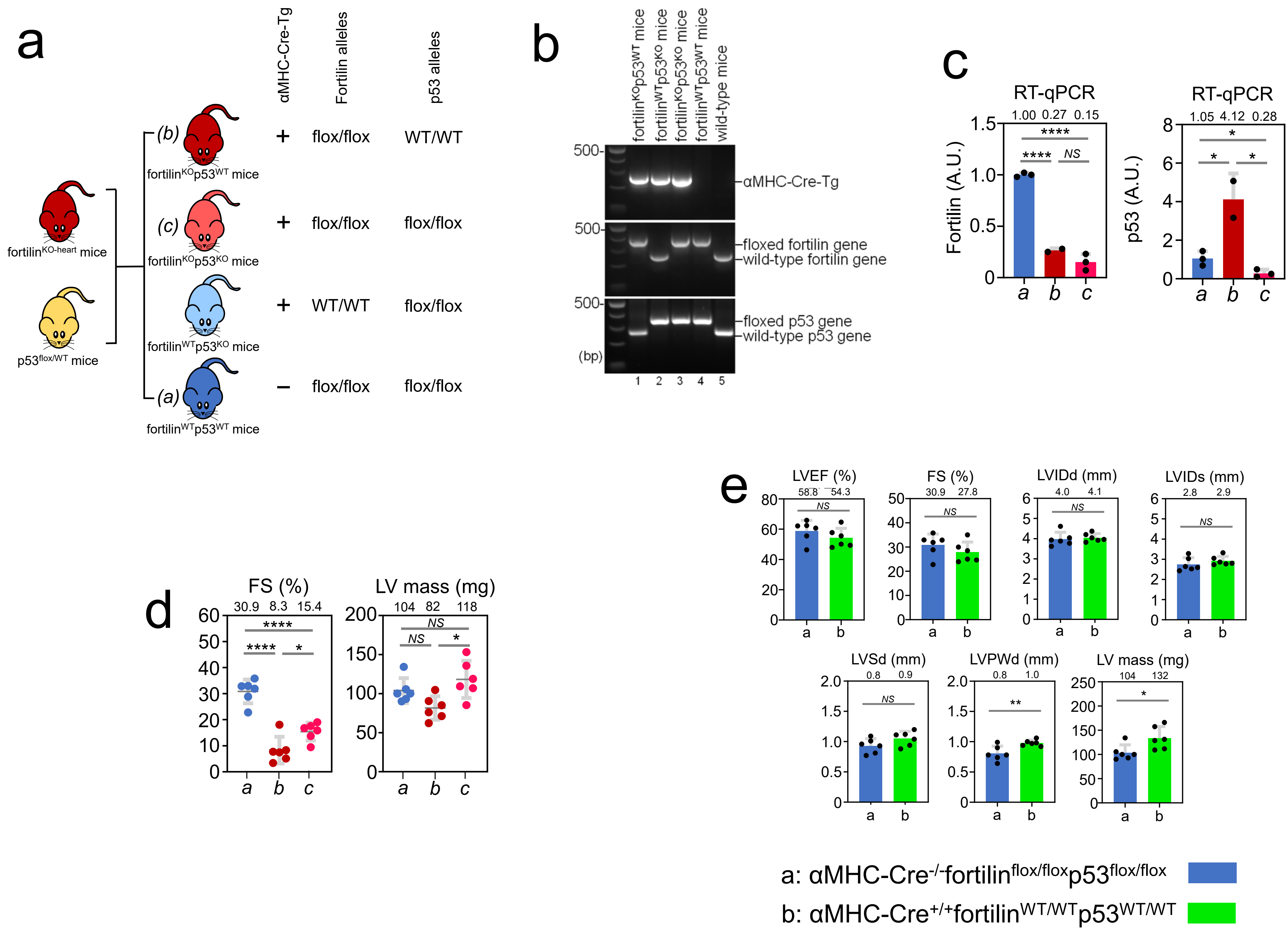
a
αMHC-Cre-Tg
Fortilin alleles
p53 alleles
fortilinKO-heart mice
p53flox/WT mice
(b) fortilinKOp53WT mice + flox/flox WT/WT
(c) fortilinKOp53KO mice + flox/flox flox/flox
fortilinWTp53KO mice + WT/WT flox/flox
(a) fortilinWTp53WT mice − flox/flox flox/flox
b
fortilinKOp53WT mice
fortilinWTp53KO mice
fortilinKOp53KO mice
fortilinWTp53WT mice
wild-type mice
500-
αMHC-Cre-Tg
floxed fortilin gene
wild-type fortilin gene
floxed p53 gene
wild-type p53 gene
(bp)
1 2 3 4 5
c
RT-qPCR
1.00 0.27 0.15
Fortilin (A.U.)
p53 (A.U.)
1.05 4.12 0.28
NS
d
FS (%)
30.9 8.3 15.4
LV mass (mg)
104 82 118
e
LVEF (%)
58.8 54.3
FS (%)
30.9 27.8
LVIDd (mm)
4.0 4.1
LVIDs (mm)
2.8 2.9
LVSd (mm)
0.8 0.9
LVPWd (mm)
0.8 1.0
LV mass (mg)
104 132
a: αMHC-Cre-/-fortilinflox/floxp53flox/flox
b: αMHC-Cre+/+fortilinWT/WTp53WT/WT

(Fig. S2)

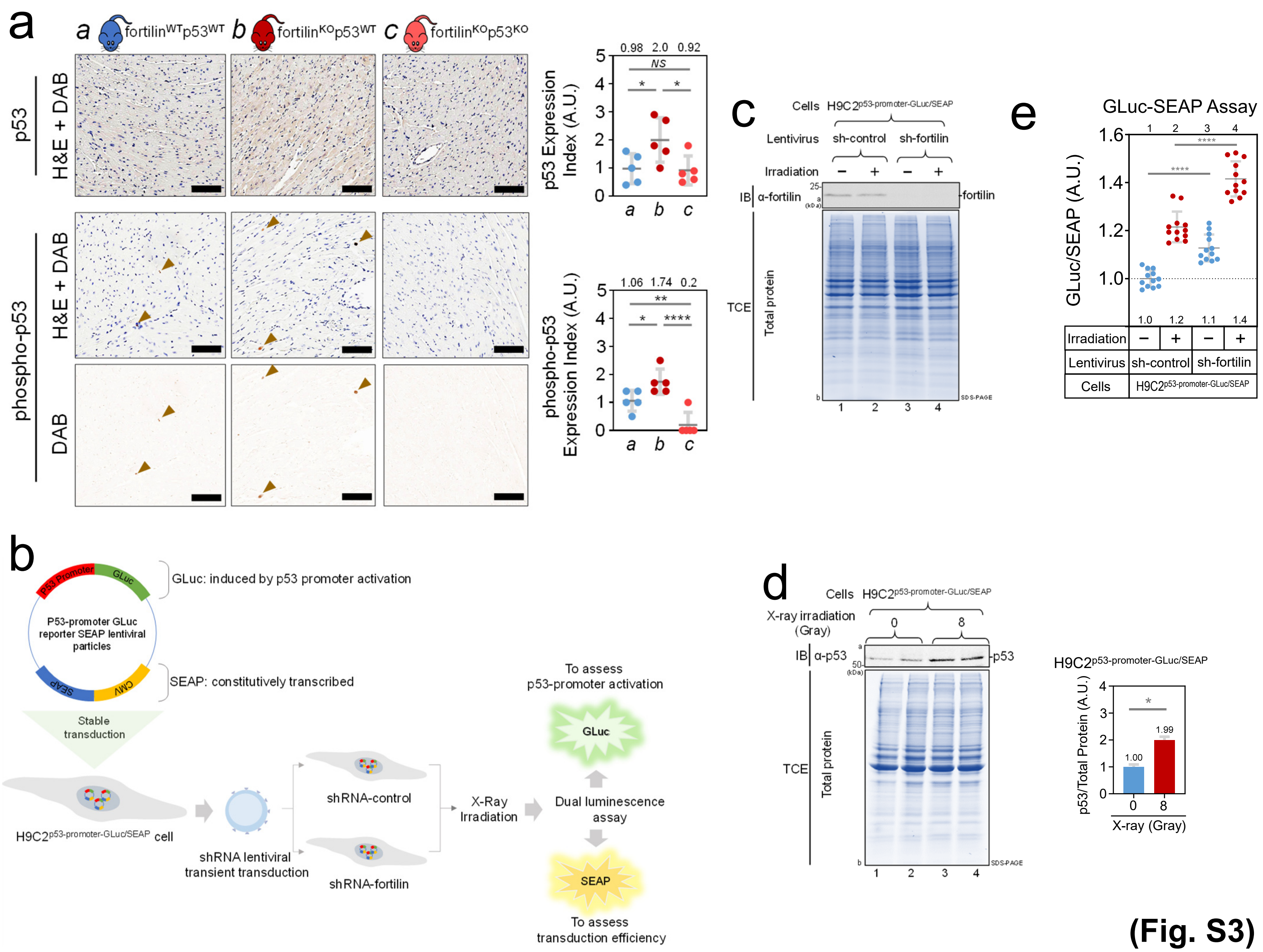
a
a fortilinWTp53WT
b fortilinKOp53WT
c fortilinKOp53KO
p53
H&E + DAB
phospho-p53
DAB
p53 Expression Index (A.U.)
0.98 2.0 0.92
NS
phospho-p53 Expression Index (A.U.)
1.06 1.74 0.2
b
GLuc: induced by p53 promoter activation
P53-promoter GLuc reporter SEAP lentiviral particles
SEAP: constitutively transcribed
Stable transduction
H9C2p53-promoter-GLuc/SEAP cell
shRNA lentiviral transient transduction
shRNA-control
shRNA-fortilin
X-Ray Irradiation
Dual luminescence assay
To assess p53-promoter activation
GLuc
SEAP
To assess transduction efficiency
c
Cells H9C2p53-promoter-GLuc/SEAP
Lentivirus sh-control sh-fortilin
Irradiation
IB α-fortilin
fortilin
TCE
Total protein
SDS-PAGE
d
Cells H9C2p53-promoter-GLuc/SEAP
X-ray irradiation (Gray)
IB α-p53
p53
TCE
Total protein
SDS-PAGE
H9C2p53-promoter-GLuc/SEAP
p53/Total Protein (A.U.)
1.00
1.99
X-ray (Gray)
e
GLuc-SEAP Assay
GLuc/SEAP (A.U.)
1.0 1.2 1.1 1.4
Irradiation
Lentivirus sh-control sh-fortilin
Cells H9C2p53-promoter-GLuc/SEAP

(Fig. S3)

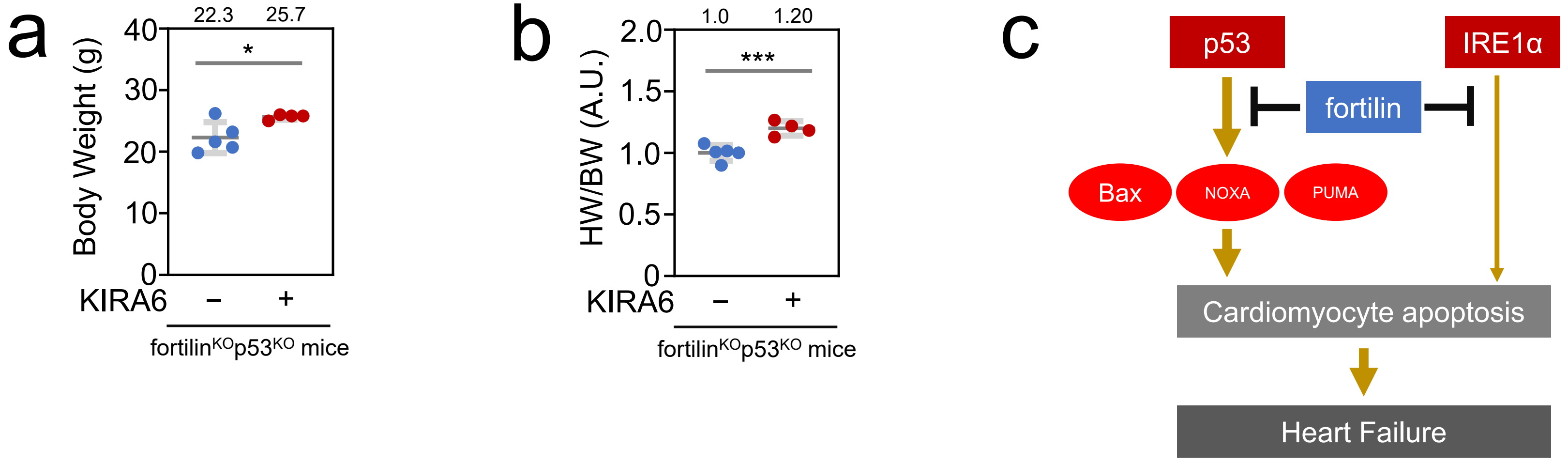
a
Body Weight (g)
22.3
25.7
*
KIRA6
−
+
fortilinKOp53KO mice
b
HW/BW (A.U.)
1.0
1.20
***
KIRA6
−
+
fortilinKOp53KO mice
c
p53
IRE1α
fortilin
Bax
NOXA
PUMA
Cardiomyocyte apoptosis
Heart Failure

(Fig. S4)
